# Reduced tonic inhibition after stroke promotes motor performance and epileptic seizures

**DOI:** 10.1038/srep26173

**Published:** 2016-05-18

**Authors:** Nadine Jaenisch, Lutz Liebmann, Madlen Guenther, Christian A. Hübner, Christiane Frahm, Otto W. Witte

**Affiliations:** 1 Hans-Berger Department of Neurology, Jena University Hospital, D-07747 Jena, Germany; 2 Institute of Human Genetics, Jena University Hospital, D-07743 Jena, Germany

## Abstract

Stroke survivors often recover from motor deficits, either spontaneously or with the support of rehabilitative training. Since tonic GABAergic inhibition controls network excitability, it may be involved in recovery. Middle cerebral artery occlusion in rodents reduces tonic GABAergic inhibition in the structurally intact motor cortex (M1). Transcript and protein abundance of the extrasynaptic GABA_A_-receptor complex α_4_β_3_δ are concurrently reduced (δ-GABA_A_Rs). *In vivo* and *in vitro* analyses show that stroke-induced glutamate release activates NMDA receptors, thereby reducing KCC2 transporters and down-regulates δ-GABA_A_Rs. Functionally, this is associated with improved motor performance on the RotaRod, a test in which mice are forced to move in a similar manner to rehabilitative training sessions. As an adverse side effect, decreased tonic inhibition facilitates post-stroke epileptic seizures. Our data imply that early and sometimes surprisingly fast recovery following stroke is supported by homeostatic, endogenous plasticity of extrasynaptic GABA_A_ receptors.

Stroke is the major cause of an acquired lifelong physical disability^1^. As a further complication, patients surviving brain ischemia often develop enhanced brain excitability and epileptic seizures, which negatively affect functional outcomes and have a considerable social and psychological impact on stroke patients^2^.

Motor impairments, including sensorimotor failures, hemiparesis, ataxia and spasticity, are the most common deficits after stroke and affect up to 80% of patients^3^. Spontaneous functional recovery frequently occurs following stroke^4^, and lesion-induced brain plasticity can be used to restore function^5^,^6^, especially if early rehabilitative training is performed^7^,^8^,^9^. Though several studies indicate the benefits of an early mobilization, an intensive training commencing too early may have a detrimental impact on stroke recovery^10^. Therefore, the optimal time to start out of bed activity should be adapted to stroke severity, age as well as frequency and time of rehabilitative interventions^11^. Unfortunately, the extent of motor recovery is highly variable between stroke patients, and the molecular basis of this form of plasticity is still unknown.

The neural basis for motor recovery following stroke depends on brain plasticity, which defines the ability of the brain to structurally reorganize and adapt its function. Brain plasticity-dependent motor learning is mainly controlled by GABA mediated inhibition^12^,^13^. GABA, the major inhibitory transmitter in the central nervous system, activates GABA_A_ receptors, which are composed of heterogeneous combinations of receptor subunits, to fine-tune fast synaptic inhibition and to control overall network excitability by tonic inhibition. In the cerebrum, the GABA_A_ receptor subunits δ and α_5_ have been identified as specific subunits incorporated into extrasynaptic GABA_A_ receptors that mediate tonic inhibition.

The potential pharmacological modulation of tonic inhibition has attracted considerable attention in stroke research. As a novel therapy, the administration of a benzodiazepine inverse agonist specific for the GABA_A_ receptor subunit α_5_ (L655,708) has been suggested to reduce excessive tonic inhibition found in the peri-infarct cortex following photothrombotic injury^14^. In light of enhanced lesion-induced plasticity^5^,^6^ and the occurrence of well-known post-stroke epilepsy^2^,^15^, the observation of increased brain inhibition following stroke is surprising.

Indeed, by screening of transcriptome data, we found a significant decrease of the extrasynaptically localized GABA_A_ receptor subunit δ following stroke in mice^16^. Stroke was induced by reversible occlusion of the middle cerebral artery in mice. The majority of strokes in humans (over 80%) are ischemic strokes resulting from blockage of blood vessels in the brain^17^. Since most ischemic strokes occur in the territory of middle cerebral artery, occlusion of this artery in mice is perfectly suitable to model focal brain ischemia in humans^18^. A decrease in tonic GABAergic inhibition following ischemic stroke may contribute to the described period of post-stroke plasticity and therefore may enhance the efficacy of rehabilitative therapies or even mediate spontaneous functional recovery^5^. Moreover, an attenuated tonic inhibition may facilitate seizures, which occur as a complication of stroke. We here studied tonic inhibition as a possible mediator of post-stroke brain hyperexcitability.

Our data indicate that following stroke the brain itself down-regulates tonic GABAergic inhibition and produces an environment, which supports brain plasticity and thereby facilitates motor improvements. In agreement with this, a decrease in tonic inhibition was identified as a reason for post-stroke epileptic seizures. As mechanism for the impaired tonic inhibition, we identified the activation of NMDA (*N*-methyl-D-aspartate) receptors by stroke-induced excitotoxicity.

## Results

### Tonic GABAergic inhibition is decreased after stroke, which correlates with a reduction of δ-GABA_A_Rs

**Figure 1:**
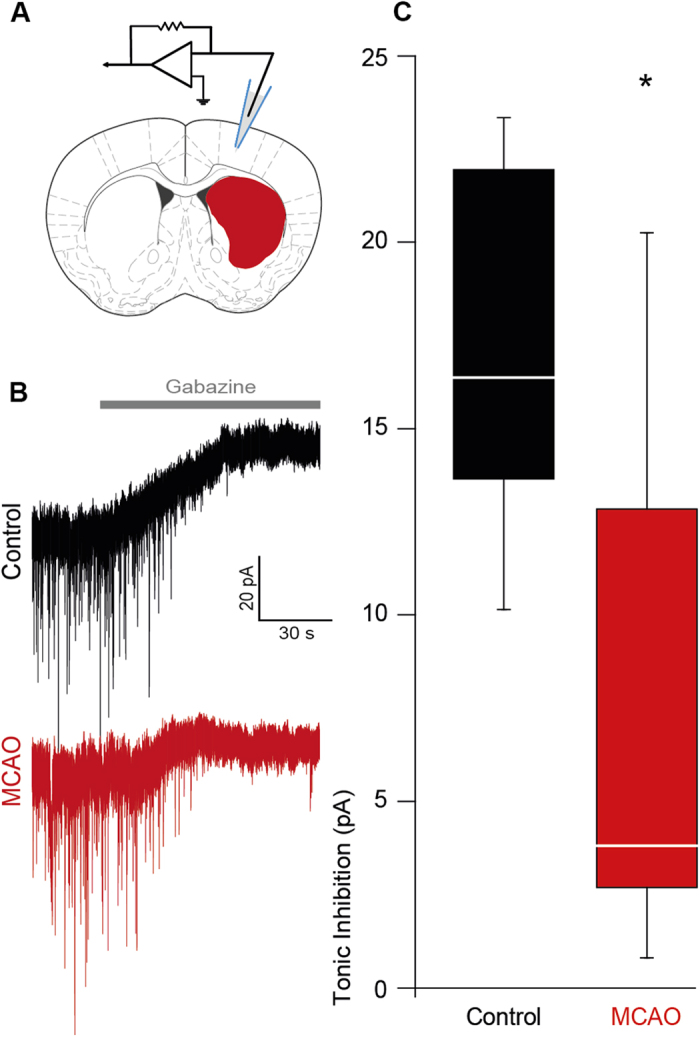
Diminished tonic inhibition in the peri-infarct cortex following stroke. **(A)** Electrode position for patch-clamp experiments, modified from The Mouse Brain in Stereotaxic Coordinates^70^. **(B)** Representative traces showing tonic inhibitory currents in control and peri-infarct neurons, respectively. **(C)** Box plot (boxes: 25–75%, whiskers: 10–90%, lines: median) showing significantly diminished tonic inhibition in the peri-infarct cortex 7 days after MCAO [MCAO, controls (cells n = 7 each, mice n = 3 each) *p ≤ 0.05].

We examined GABA_A_ receptor-mediated tonic inhibition in the peri-infarct motor cortex of mice following stroke. Occlusion of the MCA for 30 min resulted in subcortical restricted lesions surrounded by an extensive penumbra. Whole cell voltage clamp recordings were performed on layer 2/3 pyramidal neurons of the motor cortex (M1). Beside their unique morphological specificities, the input resistance (control: R_in_ 60.6 ± 7.3 MΩ; MCAO: R_in_ 77.0 ± 9.2 MΩ) was in the typical range for pyramidal cells^19^. Layer 2/3 pyramidal neurons revealed a decrease of tonic inhibition following MCAO at day 7 of reperfusion [controls: 16.37 ± 1.79 pA; MCAO: 3.81 ± 2.64 pA]. In total, 7 cells of 3 mice with stroke, and 7 cells of 3 control animals were analyzed (cells n = 7 each, mice n = 3 each, p ≤ 0.05; Fig. 1). At the contralateral side, 6 cells of 3 MCAO mice were analyzed, which were not significantly different to controls [23.01 ± 9.16 pA].

**Figure 2:**
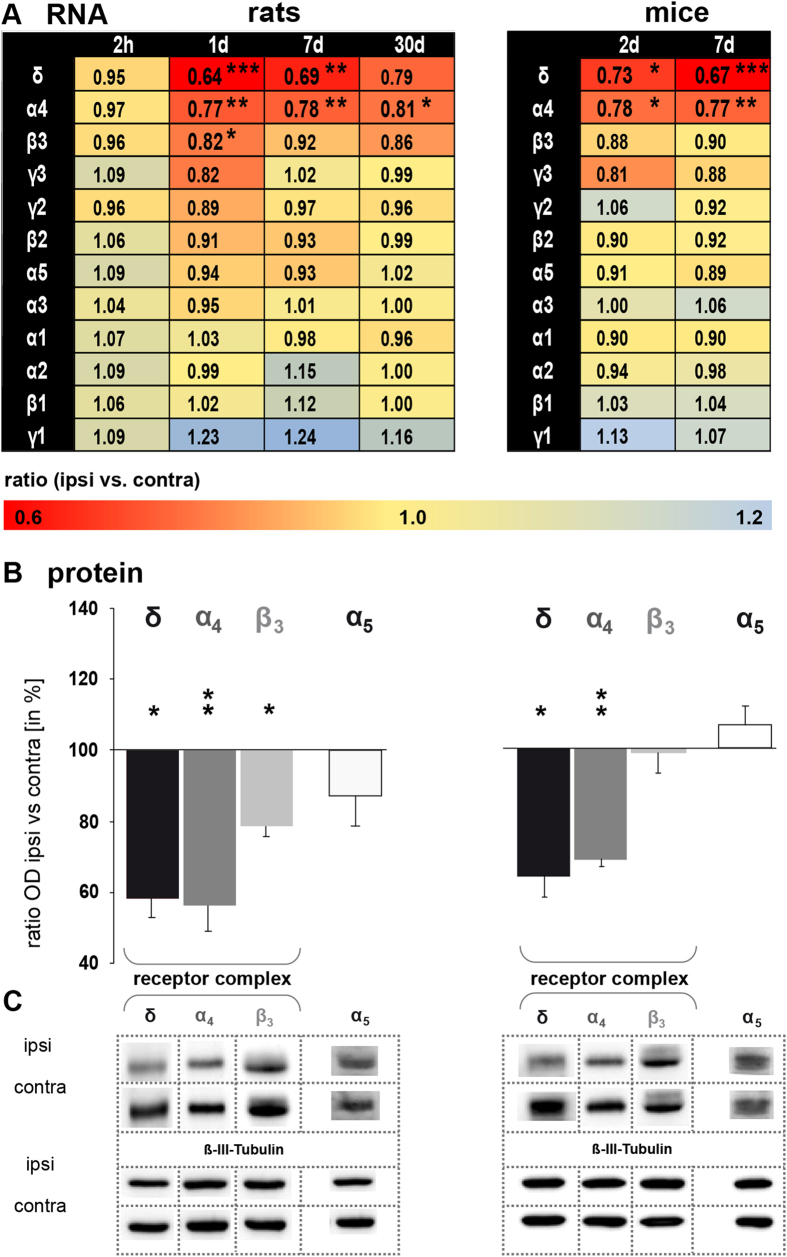
Stroke down-regulates extrasynaptically located GABA_A_ receptors containing the δ subunit. **(A)** qPCR analysis revealed a significant decrease of subunit δ, α_4_ and β_3_ transcript abundance. Transcript abundance of all other GABA_A_ receptor subunits tested remained stable. Data were normalized to *Tubb3* and displayed as the geomean of ratios ± s.e.m. [rats (n = 6), mice (n = 4), ipsi vs. contra, *p ≤ 0.05, **p ≤ 0.01, ***p ≤ 0.001]. Left column: rats; right column: mice **(B)** Western blot analysis revealed a significant decrease of subunits δ, α_4_ and β_3_ and an unaltered expression of subunit α_5_ 7 days after MCAO. Data were normalized to ß-III-Tubulin. Ratios of optical densities are shown as the percent relative to the contralateral hemisphere. Bars represent the mean ± s.e.m. [rats (n = 5), mice (n = 4), ipsi vs. contra, *p ≤ 0.05, **p ≤ 0.01]. **(C)** Representative protein bands are shown. Selected protein bands were cropped as indicated by dotted lines. All gels were run under the same experimental conditions. Ipsi- and contralateral brain samples from the same animal processed for one receptor subtype were always run on the same gel. Used antibodies and protein size of GABA receptor subunits (in kDa) are summarized in Supplementary table 1.

Reduction of tonic inhibition was paralleled by a significant down-regulation of GABA_A_ receptor subunit δ transcripts (*Gabrd*) and protein (GABRD) in peri-infarct tissue 7 days after stroke in mice [*Gabrd* ratio ipsi- vs. contralateral: 0.67 ± 0.06 (n = 4), p ≤ 0.001, and GABRD ipsi- vs. contralateral: 64.39 ± 5.93% (n = 4), p ≤ 0.05, Fig. 2A,B] as well as in rats [*Gabrd* ratio ipsi- vs. contralateral: 0.69 ± 0.10 (n = 6), p ≤ 0.01, and GABRD ipsi- vs. contralateral: 58.29 ± 5.01% (n = 5), p ≤ 0.05, Fig. 2A,B]. Expression changes of *Gabrd* were further specifically investigated in the M1 of mice, where we confirmed a significant down-regulation at 7 days after reperfusion (ratio ipsi- vs. contralateral: 0.83 ± 0.04 (n = 8), p ≤ 0.05). Reduction of *Gabrd* transcript abundance started with a delay of several hours and recovered in the subsequent four weeks (Fig. 2A). The contralateral side of sham-operated mice did not show expression differences compared to the contralateral side of MCAO mice. No change of *Gabrd* was observed in occipital brain tissue, i.e., remote from the lesion and outside of the penumbra (visual cortex at 7 days of reperfusion: ratio ipsi- vs. contralateral: 1.07 ± 0.10% (n = 5). The down-regulation of δ-subunits was further corroborated by decreased transcript and protein levels for α_4_ and β_3_, the most abundant co-subunits of δ in the cerebrum^20^ (Fig. 2A,B). Expression of subunits α_5_ was unchanged at the RNA and protein level at all time-points analyzed (Fig. 2A,B).

### Stroke-induced activation of NMDA receptors down-regulates *Kcc2* and *Gabrd*

**Figure 3:**
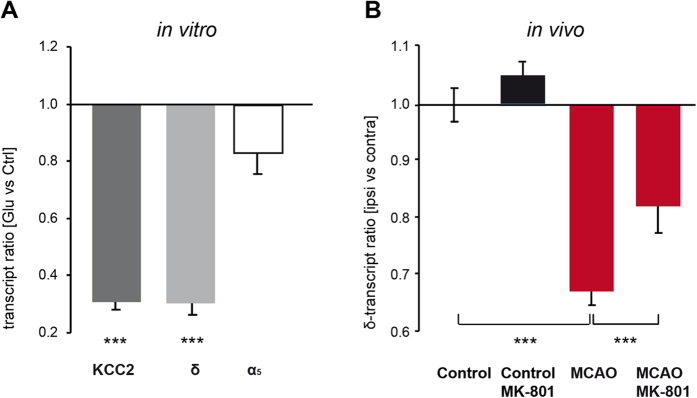
Activation of NMDA receptors by glutamate induces a down-regulation of *Kcc2* and *Gabrd* transcripts. (**A**) Application of glutamate (20 μM) to cultured cortical neurons decreased *Kcc2* and *Gabrd* but had no effect on *Gabra5*. Transcript abundance was determined one day after glutamate treatment. Data were normalized to *Tubb3* and displayed as the geomean of ratios ± s.e.m. [glutamate vs. controls, cell culture experiments (n = 6–11), ***p ≤ 0.001]. (**B**) Blocking the NMDA-receptors with MK-801 attenuated the post-ischemic down-regulation of *Gabrd*. MK-801 was injected at the onset of reperfusion. Transcript expression was determined 7 days later. Bars represent the geomean ± s.e.m. [control mice (n = 5), control + MK-801 (n = 7), MCAO (n = 9), MCAO + MK-801 (n = 7), ipsi vs. contra, ***p ≤ 0.001].

Glutamate application (20 μM) to cultured cortical neurons decreased *Kcc2* transcript abundance [ratio of glutamate (n = 11) vs. control (n = 8): 0.31 ± 0.03, p ≤ 0.001, Fig. 3A]. Likewise, *Gabrd* transcripts were reduced [ratio of glutamate (n = 11) vs. control (n = 8): 0.30 ± 0.04, p ≤ 0.001, Fig. 3A]. Glutamate treatment had no effect on the expression of *Gabra5* [glutamate (n = 7) vs. control (n = 6), Fig. 3A].

Next, we tested the pathophysiological role of excessive NMDA-receptor stimulation during stroke *in vivo*. We applied the non-competitive NMDA-receptor antagonist MK-801 to MCAO mice. Using 1 mg/kg MK-801 (given i.p. at the beginning of reperfusion), MK-801 did not affect infarct size. In control mice, MK-801 had no impact on subunit δ expression [control mice (n = 5), control + MK-801 (n = 7), Fig. 3B]. However, MK-801 significantly attenuated the post-ischemic down-regulation of *Gabrd* transcripts 7 days after MCAO [ratio ipsi- vs. contralateral MCAO: 0.65 ± 0.03 (n = 9), MCAO + MK-801: 0.81 ± 0.05 (n = 7), p ≤ 0.001 Fig. 3B]. Taken together these data suggest a glutamate-driven post-stroke mechanism of δ-GABA_A_Rs down-regulation, finally leading to decreased tonic inhibition.

### Synaptically localized GABA_A_ receptor subunits are unchanged after stroke

Heterogeneous combinations of synaptic GABA_A_ receptor subunits [α_(1–4)_, β_(1–3)_, γ_(1–3)_, typically 2α:2β:1γ]^20^,^21^ provide the basis for fine-tuning of fast synaptic GABAergic inhibition. With the present approach, we found no changes in mRNA levels of synaptically localized GABA_A_ receptor subunits (α_1_, α_2_, α_3_, β_1_, β_2_, γ_1_, γ_2_, and γ_3_) following mild MCAO [rats (n = 6), mice (n = 4), Fig. 2A). Intracellular recordings of principal neurons in the cortical layer 2/3 demonstrated that the frequency of spontaneous inhibitory postsynaptic currents (sIPSCs) was reduced, while amplitudes or receptor kinetics were unchanged (Supplementary Fig. 1), suggesting a pre-synaptic, receptor-independent change of fast synaptic inhibition.

A recent study revealed an increased phasic GABAergic signaling following permanent stroke, photothrombosis and distal MCAO. This enhanced inhibition was identified in cortical layer 5 (but not in layer 2/3) pyramidal neurons located in a small peri-lesional rim (800 μm). In accordance, the number of α1 receptor subunit containing synapses was increased in layer 5. Chronic treatment with a low dose of zolpidem, a positive allosteric modulator with high affinity for α1-GABA_A_Rs, enhanced functional recovery^22^. Unpublished data from own experiments confirm an up-regulation of α1 and other synaptically localized subunits in a small rim surrounding the lesion (cf. Figs 2 and 4 in Redecker *et al.*)^23^.

### Decreased tonic inhibition following MCAO is associated with better RotaRod performance

**Figure 4:**
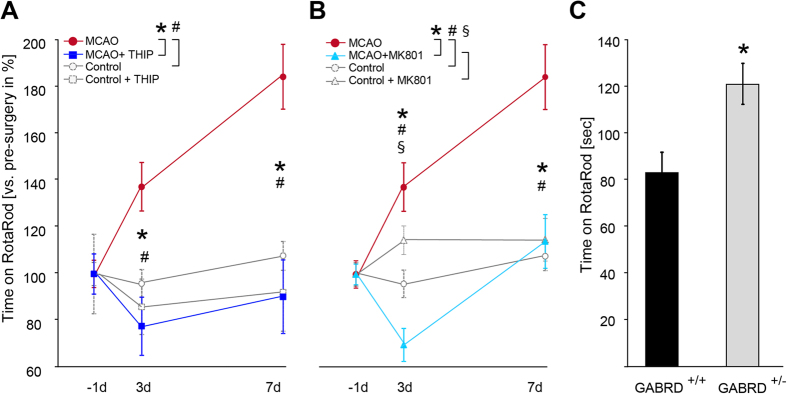
Diminished tonic GABAergic inhibition promotes post-stroke motor function. **(A)** Mice showed improved RotaRod performance 3 and 7 days after stroke [controls (n = 14), MCAO (n = 8–10), ^#^p ≤ 0.01]. Post-stroke activation of δ-GABA_A_Rs via gaboxadol (THIP) reduced motor function to control levels [MCAO (n = 8–10), MCAO + THIP (n = 8), *p ≤ 0.001]. THIP had no effect on motor performance in control mice (n = 5, Fig. 4A). Data points represent the mean ± s.e.m. [time on RotaRod vs. pre-surgery in percent]. **(B)** NMDA-receptor blockade by MK-801 reversed the improved RotaRod performance at 3 and 7 days following stroke. Bars represent the mean ± s.e.m. [control mice (n = 15), control + MK-801 (n = 14), MCAO (n = 8–10), MCAO + MK-801 (n = 12), *p ≤ 0.001, ^#^p ≤ 0.05 at 3 days, ^#^p ≤ 0.001 at 7 days, ^§^p ≤ 0.001 at 3 days). **(C)** Motor function as assessed by RotaRod was better in GABRD^+/−^ mice compared to their wild-type littermates (GABRD^+/+^). Bars represent the mean ± s.e.m. [time on RotaRod in sec, GABRD^+/+^, GABRD^+/−^ (n = 11 each), *p ≤ 0.01].

General locomotor activity after MCAO was analyzed by a walking test. Mice with MCAO displayed a significant reduced walking distance and reduced velocity compared to their controls [total distance in cm 3 days after stroke: controls (n = 5): 1435.39 ± 139.97, MCAO (n = 9): 939.98 ± 112.60, 7 days: controls: 1297.77 ± 38.50, MCAO: 914.14 ± 116.04; velocity in cm/sec 3 days: controls: 4.80 ± 0.47, MCAO: 3.14 ± 0.38; 7 days: controls: 4.33 ± 0.13; MCAO: 3.05 ± 0.39, *p ≤ 0.05]. The impact of diminished tonic inhibition on post-stroke functional motor performance was subsequently analyzed using an accelerated RotaRod test^24^, in which the mice are forced to move similar to rehabilitative training sessions to evaluate their motor coordination and balance. In our subcortical stroke model, motor performance was surprisingly strongly improved after MCAO [time on RotaRod vs. pre-surgery in percent: 3 days after stroke: 137.20 ± 10.36% and 7 days after stroke: 184.16 ± 13.81%, controls (n = 14), MCAO (n = 8–10), p ≤ 0.01; Fig. 4A]. In contrast, stroke models that destroy cortical motor regions reveal impairments on RotaRod^25^,^26^. Because the cortex was structurally intact in the present study, the impressive improvement suggests functional changes in the motor cortex, which may be related to decreased tonic inhibition. Indeed, 5 mg/kg gaboxadol (THIP), a muscimol analogue that activates extrasynaptic GABA_A_ receptors containing the δ subunit^27^,^28^, abolished the improvement of RotaRod performance in MCAO mice [3 days after stroke: 77.70 ± 12.45% and 7 days after stroke: 90.29 ± 15.72% (n = 8), Fig. 4A]. THIP had no effect on motor performance in control mice (n = 5, Fig. 4A), in agreement with previous studies^29^,^30^. Based on published data obtained from rat, subcutaneous administration of 5 mg/kg THIP induces brain levels of approximately 1,4 μM after 60 min^31^. This comparatively low concentration did not activate synaptic receptors (tested for α1β3γ_2_) but selectively agonizes α4β3δ below the superagonist behaviour which may be observed with concentrations exceeding 10 μM^32^.

Next, we analyzed the impact of the NMDA-receptor blockade on motor performance after stroke by employing the RotaRod test. MK-801 administration to MCAO mice prevented the improvement of motor performance following stroke [3 days after stroke: 69.92 ± 7.03% and 7 days after stroke: 114.05 ± 11.41 (n = 12), Fig. 4B], while it had no effect in control mice (n = 14, Fig. 4B). We conclude that the improved motor coordination early after stroke is caused by a lesion-induced plasticity mediated by the activation of NMDA-receptors with a subsequent decrease of extrasynaptic *GABA*_*A*_ receptors. Our finding that post-stroke down-regulation of δ-GABA_A_Rs is responsible for the improved motor performance could further be corroborated by the better RotaRod performance of GABRD^+/−^ vs. wild-type mice [GABRD^+/+^: 82.63 ± 8.81 sec, GABRD^+/−^: 121.12 ± 5.10 sec (n = 11 each), p ≤ 0.01; Fig. 4C].

### Reduced tonic inhibition after stroke promotes epileptic seizures

**Figure 5:**
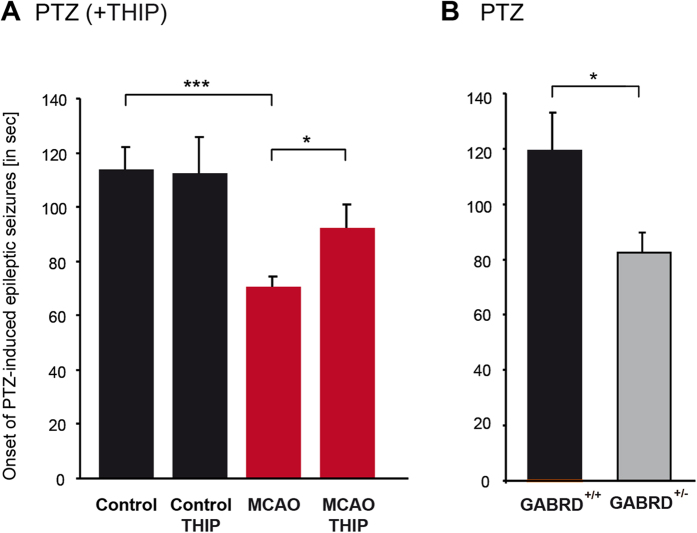
Stroke facilitates the onset of PTZ induced seizures. (**A**) Time to onset of epileptic seizures was reduced 7 days after MCAO. This effect was antagonized by gaboxadol (THIP), specifically activating extrasynaptic δ-GABA_A_Rs. THIP had no effect in control mice. Bars represent the mean ± s.e.m. [control (n = 6), control + THIP (n = 6), MCAO (n = 11), MCAO + THIP (n = 10), *p ≤ 0.05, **p ≤ 0.01]. (**B**) Time to onset of epileptic seizures was also reduced in GABRD^+/−^ mice compared to their wild-type littermates (GABRD^+/+^). Bars represent the mean ± s.e.m. [GABRD^+/+^ (n = 4), GABRD^+/−^ (n = 9), *p ≤ 0.05].

Post-stroke reduction of tonic inhibition may be expected to enhance seizure susceptibility. To test this hypothesis, we injected pentylenetetrazole (PTZ, 60 mg/kg) and determined the interval after which epileptic seizures appeared^33^. Seven days after MCAO, seizure susceptibility was significantly enhanced [controls: 113.75 ± 8.38 sec (n = 6); MCAO: 70.59 ± 3.56 sec (n = 11), p ≤ 0.01; Fig. 5A]. The post-ischemic application of 5 mg/kg gaboxadol (THIP), to activate extrasynaptic δ-GABA_A_Rs, delayed the onset of seizures in response to PTZ in MCAO mice [92.26 ± 8.83 sec, (n = 10), p ≤ 0.05; Fig. 5A) but had no effect on the onset of epileptic discharges in control mice (n = 6, Fig. 5A)^34^. The impact of extrasynaptic GABA_A_ receptors on the seizure threshold was corroborated by experiments on transgenic mice with reduced expression of the δ subunit: GABRD^+/−^ mice displayed a higher seizure susceptibility than control mice [GABRD^+/+^: 119.78 ± 13.20 sec (n = 4); GABRD^+/−^: 82.46 ± 7.35 sec (n = 9), p ≤ 0.05; Fig. 5B). These results revealed a down-regulation of extrasynaptic δ-GABA_A_Rs as a cause of post-stroke seizures.

## Discussion

### Tonic GABAergic inhibition is down-regulated following stroke

Here, we identified a decrease of tonic GABAergic inhibition following MCAO in adult mice by patch-clamp recordings in the motor cortex (M1). This reduction was paralleled by a down-regulation of the extrasynaptically localized GABA_A_ receptor subunit δ as well as by a decrease of its α_4_ and β_3_ co-subunits. We considered glutamate-driven excitotoxicity, which plays a key role in ischemic brain injury, as a potential mechanism of down-regulation^35^. Previous studies showed that NMDA receptor activity^36^,^37^, as well as stroke^38^, down-regulate the neuron-specific K^+^ Cl^−^ co-transporter KCC2, which in turn is associated with a decreased expression of δ-containing GABA_A_ receptors^39^. We tested our hypothesis that glutamate induces the down-regulation of δ-GABA_A_Rs via a KCC2-mediated mechanism in a cell culture model. Glutamate application to cortical neurons markedly decreased transcript expression of *Kcc2* and *Gabrd*. Because administration of the NMDA-receptor antagonist MK-801 to MCAO mice significantly attenuated the post-ischemic down-regulation of δ-GABA_A_Rs, an excitotoxicity-driven mechanism is corroborated *in vivo*.

Several cerebral pathophysiologies that are associated with excitotoxicity e.g., ischemia^38^,^40^, amyotrophic lateral sclerosis^41^ and epilepsy^36^, lead to down-regulation of KCC2. Mechanisms identified for KCC2 down-regulation include calpain-dependent cleavage^37^, phosphorylation of KCC2 at tyrosine 903/1087^42^ as well as dephosphorylation at serine 940^43^ and BDNF-induced TrkB activation^44^. Although most of these mechanisms point to post-translational processes, we and others have revealed an excitotoxicity-associated down-regulation of KCC2 already at the transcript level^44^. The contribution of the neuronal Cl^−^-extrusion in overall GABAergic inhibition has been extensively described, but less is known concerning KCC2 function and tonic inhibition. A recent study investigated KCC2-mediated intracellular chloride levels as possible regulators of the developmental switch in GABA_A_ receptor subunits. They found that tonic current amplitudes and the surface expression of the δ subunit decreased following KCC2 shRNA transfection^39^, which indicates a KCC2-mediated mechanism of GABRD regulation. In contrast, studies on KCC2-mutant mice (15–20% remaining cerebral KCC2 expression) suggest that tonic inhibition is not affected^45^. Here, we demonstrate a simultaneous down-regulation of both transcripts under conditions of excitotoxicity, but if and how KCC2 specifically regulates δ-GABA_A_Rs remains elusive.

After photothrombotic stroke, a model which is virtually devoid of a penumbra^6^, enhanced tonic inhibition was described in a narrow rim surrounding the lesion^14^. This enhancement of tonic inhibition is due to impaired uptake of GABA via GAT-3/GAT-4^14^. We confirmed significant decreased GAT3/4 expression in peri-infarct tissue following photothrombosis but did not detect a change in GAT3/4 expression following MCAO (Supplementary Fig. 2). The expression of *Gabrd* and *Gabra5* was unchanged following photothrombosis in several peri-lesional and remote brain regions in adult mice (Supplementary Fig. 2). Earlier work from our laboratory yielded variable results with respect to α5 subunit expression following a photothrombotic stroke: while Redecker *et al.*^23^ observed a down-regulation in the peri-lesional rat cortex, Schmidt *et al.*^46^ described non-significant changes in young rats, but an up-regulation in aged rats. The variability with respect to the regulation of α5 subunits may partially be attributed to the comparatively low expression in the cortex. *Gabra5* RNA and protein expression were unaffected following MCAO. This does not exclude that a further pharmacological reduction of tonic inhibition mediated by α5-GABA_A_Rs may augment functional recovery^14^, although at the risk of inducing epileptic activity.

In conclusion, the change of excitability may therefore depend on the type of the ischemic lesion especially with respect to glial scar formation and thus contribute to the observed clinical variability.

### Stroke-induced down-regulation of tonic inhibition is associated with a better motor performance on the RotaRod

Following MCAO, a walking test revealed decreased general motor activity but when mice were forced to move on a RotaRod at 3 and 7d following stroke they behaved better than controls. The improved RotaRod performance stands in apparent contradiction to previous results. First, enhancement in motor coordination requires a morphological intact M1, a region that is mainly involved in RotaRod training^47^. Own observations in MCAO mice with cortical damage revealed an impaired RotaRod performance following stroke (unpublished data) in agreement with other studies^25^,^48^,^49^. Second, in our model, lower post-ischemic tonic GABAergic inhibition in the M1 underlies this improved motor performance. Blockade of the excitotoxicity-mediated decrease in tonic inhibition by MK-801 or a post-stroke activation of tonic inhibition via gaboxadol (THIP) reduced RotaRod performance to control levels. Third, the RotaRod test we used provided a good grip on the rod and; similar to rehabilitative training sessions, the mice are forced to move. Our observation are further in agreement with reports that describe a period of enhanced brain plasticity following stroke^5^.

GABA is known to be intimately involved in motor learning as well as in rehabilitation following stroke. Healthy volunteers with a better performance in motor learning had a greater decrease in their GABA levels in response to transcranial direct current stimulation (tDCS)^12^. Stroke itself is known to diminish GABA, and lower levels of GABA are associated with better recovery in stroke patients^50^. Therefore, the ability to decrease cortical GABA is important to facilitate motor skills in humans. The post-stroke decrease in GABA was localized to the ipsilateral M1^50^, the cortical region where we identified a decrease in δ-subunits in association with attenuated tonic inhibition. Decreased GABA in M1 might be an additional source of impaired tonic inhibition.

While several studies point to an advantage of a decrease in GABAergic inhibition to facilitate motor skills, the specific relevance of tonic inhibition in this context remains uncertain. Blocking of excessive tonic inhibition was beneficial for the recovery of motor functions following photothrombotic stroke^14^ and suppressed hyperactivity in an animal model of Down syndrome^51^. In both studies, the effect was mediated by negative allosteric modulators (or benzodiazepine-site inverse agonists) selective for α5-GABA_A_Rs. Following a unilateral lesion of the anteromedial cortex, ipsilateral muscimol infusions disrupted the recovery from somatosensory asymmetry^52^. Muscimol acts as a super agonist on δ-GABA_A_Rs with increased efficacy compared to GABA^53^. In contrast, the potentiation of tonic inhibition via gaboxadol (THIP, a muscimol analogue) reduced cerebellar ataxia in an animal model of Angelman syndrome^54^ and attenuated motor hyperactivity in the mouse model of Fragile X Syndrome^55^. Moreover, an increase in extrasynaptic GABA is involved in the control of motor excitability observed in Tourette syndrome^56^.

These studies revealed the tight homeostatic control of motor functions by GABAergic tonic inhibition because variations above and below trigger motor dysfunctions. In our model, decrease of tonic inhibition facilitates motor improvements. We therefore suggest that higher cortical excitability induces a homeostatic plasticity that can be used for rehabilitation. Whereas this is in agreement with the accepted role of GABA in controlling excitability, the specific impact of GABAergic tonic inhibition on the emerging post-stroke motor plasticity is new. Moreover, we suggest that the regulation of tonic inhibition following stroke might be a reason for the inter-individual differences in behavioral performance and recovery. This is supported by our results that reveal an attenuated decrease of *Gabrd* following stroke in aged vs. young mice^16^, which suggests a reduced cortical plasticity and less frequent spontaneous recovery in the elderly.

### Stroke facilitates the onset of epileptic seizures

Patients surviving brain ischemia often develop enhanced brain excitability and epileptic seizures^2^. Post-stroke seizures are treated like epilepsy with all the negative side effects of anticonvulsants like a harmful impact on functional recovery, cognition, bone health and interference with other medications. Therefore negative effects of anti-epileptic drugs stand against the potential benefits of seizure reduction.

We therefore studied tonic inhibition as a possible mediator of post-stroke brain hyperexcitability. PTZ administration promoted the onset of epileptic seizures in mice after MCAO. This effect was abolished by the post-ischemic application of gaboxadol (THIP). We also confirmed the impact of lower tonic inhibition on seizure thresholds in GABRD^+/−^ mice, which is consistent with the findings of previous studies on GABRD^−/−^ mice^57^.

Missense mutations within the human GABRD gene tend to decrease GABA_A_R function and are found to be associated with generalized idiopathic epilepsies^58^,^59^,^60^. Therefore, it is not surprising that ambient GABA and extrasynaptically localized GABA_A_Rs are targets of anticonvulsant drugs^61^. Although we identified post-stroke epileptic seizures as THIP sensitive, we cannot exclude the down-regulation of KCC2 as a further cause of enhanced brain activity. Its knockdown in mice increases neuronal excitability, and rare variants of KCC2 confer an increased risk of epilepsy in men^62^.

Here, we identified a decrease in GABAergic tonic inhibition as a cause of epileptic seizures following stroke. This is important because it opens the possibility of specifically treating severe post-stroke seizures and thereby omitting the side effects that often emerge from anticonvulsants. Gaboxadol (THIP), a muscimol derivative, is an attractive candidate in this context.

Survivors of stroke often recover from motor deficits, either spontaneously or during early rehabilitative training^4^,^7^,^8^,^9^. Unfortunately, the extent of motor recovery is highly variable between stroke patients as well as their responsiveness to rehabilitative therapies. We identified decreased GABAergic tonic inhibition as the molecular basis of post-stroke plasticity. The regulation of tonic inhibition following stroke might be a reason for the inter-individual differences in patient recovery and the occurrence of epileptic seizures. Therefore, our findings have important implications for the rehabilitation of stroke patients.

## Methods

### Animals

All studies were performed on 2–3-month-old male Wistar rats and C57Bl/6J mice. All animal care and experimental procedures were performed in accordance with EU animal welfare protection laws and regulations. We confirm that all experimental protocols were approved by a licensing committee from the local government (Landesamt für Verbraucherschutz, Bad Langensalza, Thuringia, Germany). GABA_A_ receptor subunit δ - deficient mice; GABRD^+/− ^63 were kindly provided by W. Sieghart, University of Vienna, Austria.

### Transient MCA occlusion (MCAO)

MCAO was performed as described in detail for rats^64^ and mice^65^. Occlusion of the MCA was performed for 30 min. The protocol of a mild MCAO we applied here produces infarcts which are mainly restricted to the territory of the MCA and therefore affect the striatum, but does not damage the thalamus. The rat brains were removed after cervical dislocation at two hours, one day, 7 and 30 days after reperfusion. Using a rat brain slicer (Rodent Brain Matrix, Adult Rat, Coronal Sections; ASI Instruments, USA), coronal sections (3 mm) comprising the infarct (bregma 1.0 to −2.0 mm) were dissected and subsequently separated into the ipsi- and contralateral hemisphere. The mice were sacrificed by cervical dislocation with subsequent brain removal on day 2 and 7 after reperfusion. Using a Precision Brain Slicer (BS-2000C Adult Mouse; Braintree Scientific Inc., USA), coronal sections (2 mm) comprising the infarct (bregma +0.8 to −1.2 mm) were separated into the contra- and ipsilateral hemisphere, hereafter referred to as peri-infarct tissue.

### Photothrombotic lesions

Photothrombosis was induced in anaesthetized mice by fixing the head in a stereotactic frame. A fiberoptic bundle (diameter 1.3 mm) connected to a cold light source (KL 1500; Schott, Germany) was positioned in the middle between the bregma and lambda, 1.5 mm lateral to the mid-sagittal suture. Following Bengal Rosa administration into the tail vein (50 mg/kg body weight), the skull was illuminated for 15 min. The controls underwent the same procedure but without illumination. The brains were removed after cervical dislocation 7 days following injury induction. Specific brain regions were dissected.

### Slice preparation and patch-clamp recordings

Patch-clamp recordings were performed as described previously^66^. Coronal brain slices of mice were prepared 7 days after MCAO. Briefly, cortical layer 2/3 neurons were patched using a CsCl-based intracellular solution (in mM): 122CsCl, 8NaCl, 0.2MgCl_2_, 10HEPES, 2EGTA, 2Mg-ATP, 0.5Na-GTP, pH adjusted to 7.3 with CsOH in the whole-cell modus at a holding potential of −70 mV. DL-APV (30 μM), CNQX (10 μM) and GABA (5 μM) were added to aCSF. Total GABAergic currents consisting of both phasic and tonic components were blocked by the application of 100 μM gabazine. The size of the tonic inhibitory current was determined by subtracting the holding current under baseline conditions from the holding current in the presence of gabazine. Holding currents were estimated by plotting 20 sec periods of raw data in an all-points histogram (Clampfit 10.2; Molecular Devices). A Gaussian line was fitted to the right part of the smoothed curve (Savitzky-Golay algorithm), which was not skewed by synaptic events. The mean of the fitted Gaussian line was considered the mean holding current^67^. Recordings of sIPSCs were performed using the same intracellular solution as for the determination of the tonic inhibition. DL-APV (30 μM), CNQX (10 μM) and GABA (5 μM) were added to the perfusate. The currents were identified as events when the rise time was faster than the decay time. The following parameters were determined: inter-event interval, frequency, rise time, peak amplitude and the time constant of decay (τ). The decay of each event was fitted with a monoexponential curve in pClamp. Only residual SDs <0.3 were accepted as a criterion for the quality of the fit.

### Quantitative PCR (qPCR)

Total RNA was isolated using the RNeasy Lipid Tissue Mini Kit (Qiagen, Germany) or using the phenol/chloroform extraction method. qPCR was performed as described previously^65^. The specific primers are listed in Supplementary Table 1. qPCR was performed in a 20 μl amplification mixture consisting of Brilliant^®^ II or III SYBR Green QPCR Master Mix (Stratagene, USA), cDNA (equivalent to 25 ng reverse-transcribed RNA) and primers (at a final concentration of 250 nM each). Specific transcripts were amplified with Rotor Gene 6000 (LTF-Labortechnik/Corbett Life Science, Australia). Expression of each GABA_A_ receptor subunit was normalized to Gapdh as well as to Tubb3. The relative expression levels (MCAO ipsi vs. contra and MCAO contra vs. sham contra) were calculated using the Pfaffl equation^68^.

### Western blot analyses

Protein isolation and Western blotting were performed as described previously^38^. Whole proteins were isolated and equal amounts of protein (10 μg each) were applied. The antibodies are listed in Supplementary Table 1. Proteins were detected by chemiluminescence (Immobilon Western Chemiluminescent HRP Substrate; Millipore, Germany) and analyzed with the image acquisition system LAS 3000 (Fujifilm, Japan). The optical density of each protein band was quantified with AIDA Image Analyzer Software (Raytest, Germany). Optical densities were normalized to ß-actin as well as to ß-III-Tubulin.

### Neuronal cell culture

Cortical neurons were dissected from mouse embryos as described previously^66^,^69^. In brief, brain tissues from 2–3 embryos (E18^+/−^1day) were removed and transferred into fresh 1× Hanks’ balanced salt solution on ice (HBSS, Gibco). Dissected cortices were rinsed with HBSS and were trypsinized with 0.25% trypsin at 37 °C for 30 min. The supernatant was removed, and the cortices were washed with HBSS and passed through Pasteur pipettes of decreasing diameter to dissociate the tissue. Approximately 250,000 cells were plated onto poly-L-lysine (Sigma-Aldrich)-coated coverslips (ø 30 mm). Cells were allowed to settle for 120 min at 37 °C and 5% CO2 in the incubator. Finally, coverslips were transferred to 6 cm culture dishes containing Neurobasal medium (Invitrogen) supplemented with 1 mM L-glutamine, B27 supplement (Gibco), and penicillin and streptomycin (Gibco). Medium was added once a week, and the cultures were maintained for 3 weeks before the experiments. At DIV17–18, cells were incubated with 20 μM glutamate for 10 min. Twenty-four hours later, cells were harvested in Qiazol (3 coverslips each were pooled as one RNA sample) and processed for RNA isolation and qPCR as described above.

### Pharmacological interventions *in vivo*

Tonic inhibition was modulated by the GABA_A_ receptor agonist gaboxadol (also known as 4,5,6,7-tetrahydroisoxazolo(5,4-c)pyridin-3-ol THIP, Tocris Bioscience, UK). THIP was administered i.p. at a dose of 5 mg/kg BW 30 min before starting the RotaRod tests and 30 min before epilepsy induction. MK-801, a non-competitive antagonist of NMDA- receptors, was administered i.p at a dose of 1 mg/kg BW at the beginning of reperfusion. Control groups received physiological saline.

### Behavioral studies

A walking task was performed to assess the locomotor activity of mice 3 and 7 days following MCAO. The walking apparatus consisted of a Plexiglas box equipped with a 12-mm square wire mesh with a grid area of 40 × 40 cm, 10 cm above the ground. A Panasonic Color CCTV Camera (Model No. WV-CP 450)/G) was fixed above the walking grid. Each mouse was placed individually on top of the grid and allowed to walk for 5 min. To assess the total distance, the moving time and the velocity, the Ethovision XT 6.1.326 software (Noldus Information Technology, Wageningen, The Netherlands) was used. Motor performance was tested by an accelerated RotaRod procedure 3 and 7 days after MCAO. The RotaRod apparatus (3375-4R; TSE Systems, Germany) consisted of a striated rod providing a good grip (diameter: 3 cm), separated in four compartments (width: 8.5 cm) and located 27.2 cm above the floor grid. One day before MCAO induction, mice were placed on the rod for 30 sec without rotation followed by 120 sec of low speed rotation at 4 rpm. Subsequently, the mice were tested in 3 trials for 5 min (4–40 rpm). After MCAO, the mice were tested under the same conditions at 3 and 7 days of reperfusion. At each trial, the latency to fall was recorded.

### Induction of epileptic seizures

Post-ischemic seizures were induced in mice 7 days after MCAO using PTZ (Sigma-Aldrich, USA). PTZ was administered i.p. at 60 mg/kg BW. Immediately after PTZ injection, the mice were placed into a video-monitored Plexiglas cage, and the convulsive behavior was observed for a period of 15 min. Native and sham-operated mice underwent the same procedure. Tonic inhibition was modulated by gaboxadol (THIP), administered i.p. at a dose of 5 mg/kg BW 30 min before PTZ injection. The control groups received physiological saline injections.

### Statistical analyses

qPCR data and the parameters of the behavioral studies were analyzed with one- or two-way analysis of variance (ANOVA) with post-hoc Tukey’s test or with Wilcoxon’s test. For statistical analysis of the patch-clamp recordings, the Mann-Whitney test, the Kolmogorov-Smirnov test or Student’s t-test was used. The onset of seizures was analyzed using the Mann-Whitney test. For Western-blot analysis, the paired Student’s t-test was used. All data are expressed as the mean or geomean ± s.e.m. The levels of significance were set at *p ≤ 0.05, **p ≤ 0.01 and ***p ≤ 0.001.

## Additional Information

**How to cite this article**: Jaenisch, N. *et al.* Reduced tonic inhibition after stroke promotes motor performance and epileptic seizures. *Sci. Rep.* **6**, 26173; doi: 10.1038/srep26173 (2016).

## Supplementary Material

Supplementary Information
