# Supplementary material for: Reduced tonic inhibition after stroke promotes motor performance and epileptic seizures
Source: Sci Rep. 2016 May 18;6:26173. doi: 10.1038/srep26173 (PMC4870642; doi:10.1038/srep26173)
Supplement: Supplementary Information [file srep26173-s1.pdf]

Supplementary information

Title: Reduced tonic inhibition after stroke promotes motor performance and epileptic seizures

Authors: Nadine Jaenisch [1], Lutz Liebmann [2], Madlen Guenther [1], Christian A. Hübner [2], Christiane Frahm [1‡*] Otto W. Witte [1‡*]

Supplementary Table 1: Primers and antibodies

**Primers for qPCR of rat brain samples**			
**mRNA**	**NM number**	**Sequence 5'→ 3'**	**Product bp**
**Gapdh**	NM_017008	Fwd: GCATTGCTCTCAATGACAACTT<br>Rev: GGCCTCTCTCTTGCTCTCAGT	162
**Tubb3**	NM_139254.2	Fwd: GGCAACTATGTGGGGGACT<br>Rev: GCACCACTCTGACCGAAGAT	191
**Gabra1**	NM_183326.2	Fwd: GATGGCAAAAGCGTGGTTC<br>Rev: TCGGTTCTATGGTCGCACTT	160
**Gabra2**	NM_001135779.1	Fwd: CAGCGAGAACTGTGTTTGGA<br>Rev: CCACTTTGGGAAGGGAATTT	84
**Gabra3**	NM_017069.1	Fwd: TGGTCATGTTGTTGGGACAG<br>Rev: TGGCAAGTAGGTCTGGATGA	118
**Gabra4**	NM_080587.3	Fwd: AGGAGTCTGTTCCAGCCAGA<br>Rev: AAAGAATGCCGAGCACTGAT	85
**Gabra5**	NM_017295.1	Fwd: CAGACGTACCTTCCCTGCAT<br>Rev: GGTTGTCATGGTCAGCACTG	120
**Gabrb1**	NM_012956.1	Fwd: CAAGACCAGAGTGCCAATGA<br>Rev: CCAGGGTGCTGAGGAGAATA	88
**Gabrb2**	NM_012957.2	Fwd: GTCAACAAGATGGACCCACA<br>Rev: GAGGCATCATAGGCAAGCAT	128
**Gabrb3**	NM_017065.1	Fwd: GACAGCCAAGGCCAAGAAT<br>Rev: TGAACATCCATCGGTGCTAG	90
**Gabrg1**	NM_080586.1	Fwd: AACCACCAGAGACAGGAAGC<br>Rev: TTCCCCTTGAGGCATAGAAA	106
**Gabrg2**	NM_183327.1	Fwd: TGTCCTGGGTATCCTTCTGG<br>Rev: AGAGACTTCCGGGCTATGGT	112
**Gabrg3**	NM_024370.3	Fwd: TCCCCTGCATACTGACTGTG<br>Rev: CGTGGTGATGCCTAATGTTG	92
**Gabrd**	NM_017289.1	Fwd: AGAAACGGAAAGCCAAGGTC<br>Rev: CCTCCTTCTTTGCCTCCACT	189
**Primers for qPCR of mouse brain samples**			
**mRNA**	**NM number**	**Sequence 5'→ 3'**	**Product bp**
**Gapdh**	NM_008084.2	Fwd: CAACAGCAACTCCCACTCTTC<br>Rev: GGTCCAGGGTTTCTTACTCCTT	164
**Tubb3**	NM_023279.2	Fwd: GCCTTTGGACACCTATTCAGG<br>Rev: ACTCTTTCCGCACGACATCT	133
**Gabra1**	NM_010250.4	Fwd: GATGGCAAAAGCGTGGTTC<br>Rev: TCGGTTCTATGGTCGCACTT	160
**Gabra2**	NM_008066.3	Fwd: TTGGGACGGGAAGAGTGTAG<br>Rev: TGGCTTGTTCTCTGGCTTCT	184

**Gabra3**	NM_008067.4	Fwd: GCCGTCTGTTATGCCTTTGT<br>Rev: CCTTGGCCAGATTGATAGGA	199
**Gabra4**	NM_010251.2	Fwd: CCCATGAGACTGGTGGATTT<br>Rev: ACAGTCTGCCCAATGAGGTC	176
**Gabra5**	NM_176942.4	Fwd: AAGAAAGCCCTGGAAGCAG<br>Rev: GTTTGGAGGATGGGTCAGC	105
**Gabrb1**	NM_008069.4	Fwd: CAAGACCAGAGTGCCAATGA<br>Rev: TGGTCTCGTTCCTGATTTCC	106
**Gabrb2**	NM_008070.3	Fwd: TGCCAACAATGAGAAGATGC<br>Rev: CCCATTACTGCTTCGGATGT	114
**Gabrb3**	NM_008071.3	Fwd: ACAATCCTCTCGTGGGTGTC<br>Rev: GAGTCTCCCGAAGGTGAGTG	118
**Gabrg1**	NM_010252.4	Fwd: TGGAATACGGAACCTTGCAT<br>Rev: TGCTGTTCATGGGAATGAGA	132
**Gabrg2**	NM_008073.2	Fwd: GGGCTACTTCACCATCCAGA<br>Rev: GACCTTGGGCAGAGATTTTC	172
**Gabrg3**	NM_008074.2	Fwd: GAAGACTCCCCATCAAACCA<br>Rev: ATTCCAATGTCCGGTCTCAG	122
**Gabrd**	NM_008072.2	Fwd: CCACTTCAATGCCGACTACA<br>Rev: TGAGAGGGAGAAAAGGACGA	106
**Antibodies for Western blotting**			
**Ab**	**Dilution**	**Company**	**kDa**
**ß-Actin**	1:90.000	beta-actin; ab8227, Abcam, UK	42
**TUJ1**	1:90.000	Neuronal Class III ß-Tubulin; TUJ1, Covance, USA	51
**GABRD**	1:250	$GABA_A$ Rδ; sc25705, Santa Cruz Biotechnology, USA	51
**GABRD**	1:500	$GABA_A$ subunit δ, kindly provided by Werner Sieghart, Center for Brain Research, Medical University Vienna, Austria	51
**GABRA5**	1:2000	$GABA_A$ receptor α5 antibody, [1]	52
**GAT-3**	1:5000	Rabbit anti-GABA transporter-3 (GAT-3) polyclonal antibody, AB1574, Millipore, Germany	70
**goat @ rbt IgG**	1:5000	HRP-conjugated goat anti-rabbit IgG antibody; 1:5000, sc-2004; Santa Cruz Biotechnology, USA	
**goat @ mouse IgG**	1:5000	HRP-conjugated goat anti-mouse IgG antibody; 1:5000, sc-2002; Santa Cruz Biotechnology, USA	
**goat @ guinea pig IgG**	1:5000	HRP-conjugated goat anti-guinea-pig IgG antibody; 1:5000, sc-2438; Santa Cruz Biotechnology, USA	

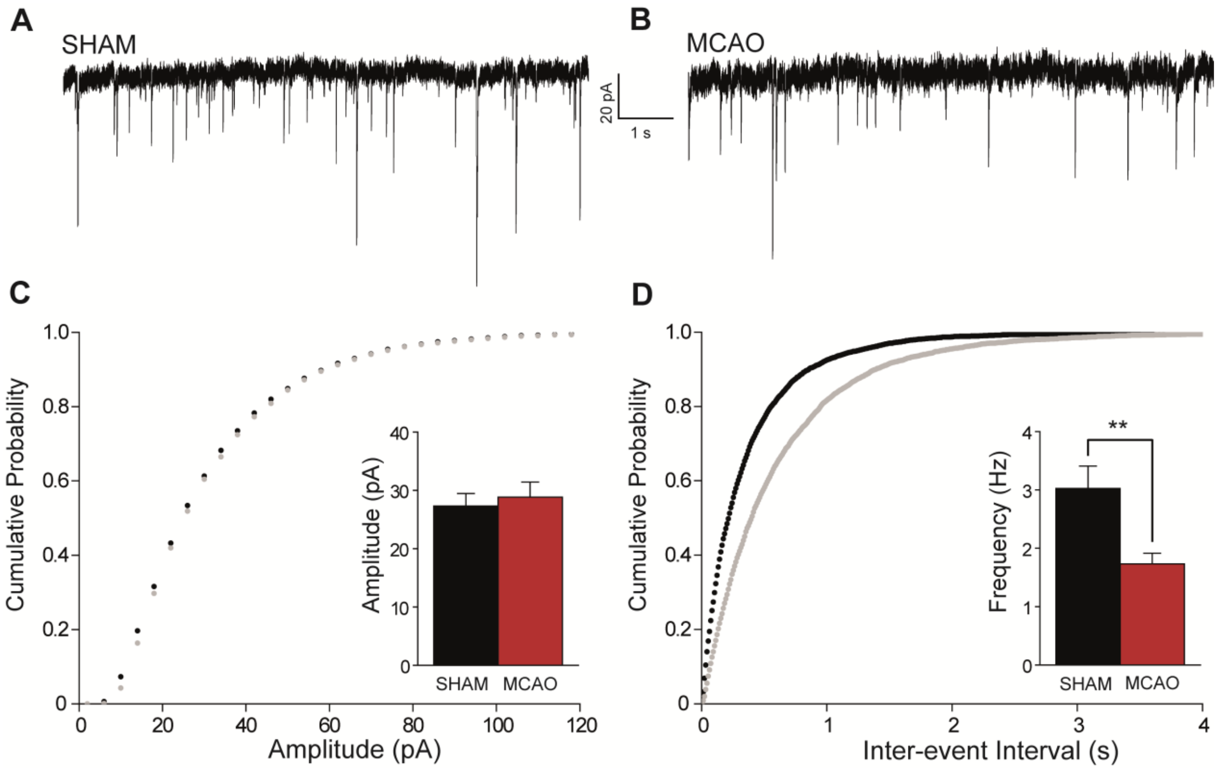

Supplementary Fig. 1. Synaptic GABAergic inhibition 7days after stroke. The frequency of sIPSCs, but not the amplitude, decreased in cortical neurons of MCAO-treated mice. **(A, B)** Representative traces of a sIPSC recorded in cortical neurons (layer 2/3) of control and MCAO mice. **(C)** Cumulative plots and the means of sIPSC amplitudes did not differ between the groups (cells n=10/12, mice n=3). **(D)** Cumulative plots of inter-event intervals and means of frequencies revealed a shift to longer intervals in neurons of the ischemic cortex (cells n=10/12, mice n=3, **p≤0.01).

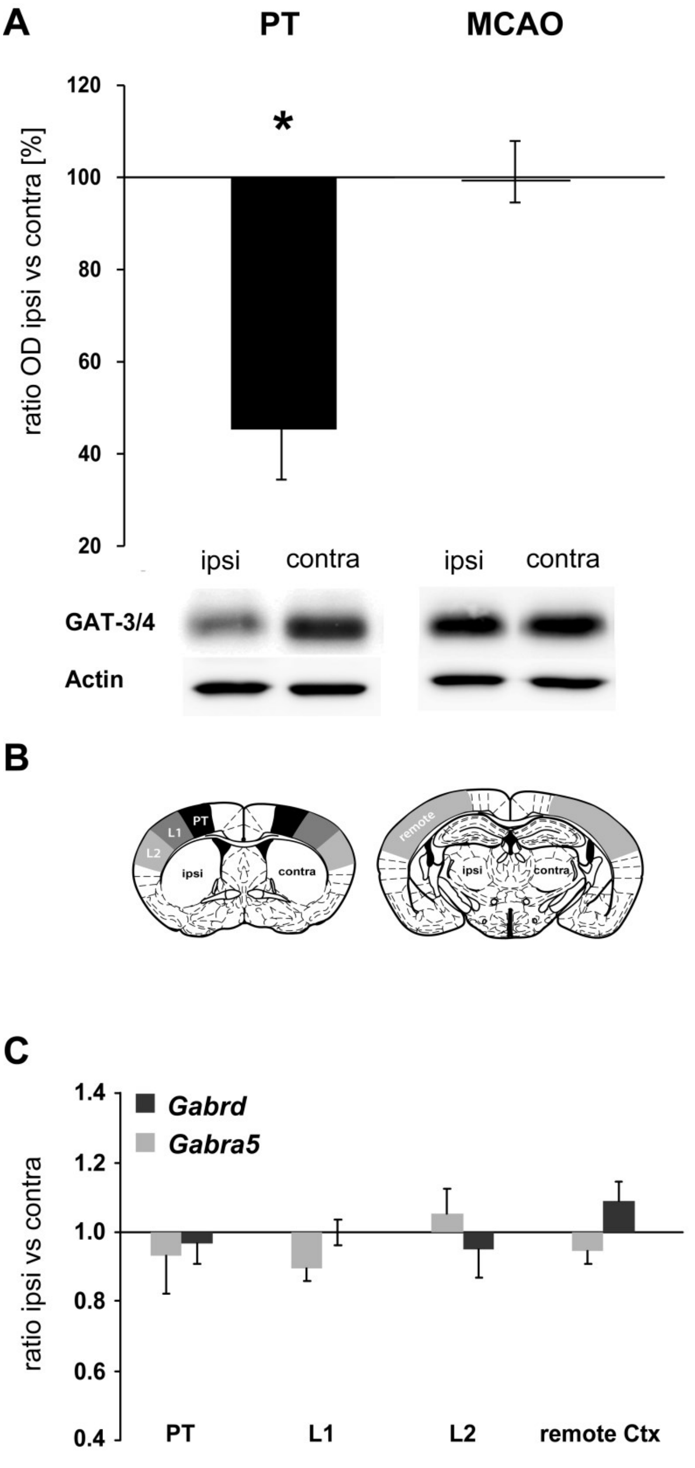

Supplemetary Fig. 2. GABA transporter GAT3/4 and $GABA_A$ receptor subunits *Gabrd* and *Gabra5* at 7days following stroke in mice. (A) GAT3/4 was reduced following photothrombosis but remained stable following MCAO. Optical densities of GAT3/4 in a ratio to ß-actin are diagrammed as the percent relative to the contralateral hemisphere±s.e.m (PT: n=3, MCAO: n=4, $*p \leq 0.05$). **(B)** Scheme of analyzed brain areas (PT: injured tissue inclusive glial scar; L1, L2: perilesional lateral regions and remote cortical area), modified from The Mouse Brain in Stereotaxic Coordinates[2]. **(C)** Normalization of *Gabrd* and *Gabra5*

to *Tubb3* showed stable post-photothrombotic RNA expression. Data are displayed as the geomean of ratios (ipsi vs. contra)±s.e.m. (PT: n=5).

1. Fritschy, J.M. & Mohler, H. GABAA-receptor heterogeneity in the adult rat brain: differential regional and cellular distribution of seven major subunits. *J Comp Neurol* **359**, 154-194 (1995).

2. Paxinos, G. & Franklin, K.B.J. *The Mouse Brain in Stereotaxic Coordinates* (Academic Press, San Diego, 2001).
